# Development of an LC-MS/MS-Based Analytical Method, In Vitro Characterization, and Pharmacokinetic Study of Rafoxanide Nanosuspension in Sheep

**DOI:** 10.3390/ani16071065

**Published:** 2026-03-31

**Authors:** Kairui Sun, Bing Li, Fusheng Cheng, Yaxin Zhou, Guonian Dai, Haiquan Li, Ligang Yuan, Jiyu Zhang

**Affiliations:** 1 College of Veterinary Medicine, Gansu Agricultural University, Lanzhou 730070, China; skrskrskr123@sina.com (K.S.); dai940910@163.com (G.D.); 2 Key Laboratory of Veterinary Pharmaceutical Development, Lanzhou Institute of Husbandry and Pharmaceutical Sciences, Chinese Academy of Agricultural Sciences, Ministry of Agriculture and Rural Affairs, Lanzhou 730050, China; libing@caas.cn (B.L.); chengfusheng@126.com (F.C.); zhouyaxin@caas.cn (Y.Z.); lihaiquan1127@163.com (H.L.)

**Keywords:** rafoxanide, nanosuspension, characterization, dissolution, LC-MS/MS, pharmacokinetics, sheep

## Abstract

This study aimed to establish a more sensitive and reliable quantitative method for determining rafoxanide (RFX) in ovine plasma. The method was applied in an 84-day long-term comparative pharmacokinetic trial to evaluate the performance of a rafoxanide nanosuspension (RFX-NS) versus its conventional suspension (RFX-S) in sheep. Characterization of the two formulations revealed the following results: RFX-NS had an average particle size of 484.93 ± 43.11 nm, a uniform size distribution (PDI 0.06 ± 0.07), and a Zeta potential of −43.59 ± 0.67 mV, which were significantly superior to those of RFX-S (particle size 2379.67 ± 121.71 nm, PDI 0.93 ± 0.10, Zeta potential −38.10 ± 0.55 mV), demonstrating excellent physical stability. In vitro dissolution tests indicated a higher dissolution rate for RFX-NS (97.6% at 60 min). By integrating formulation characterization, in vitro dissolution assessment, and pharmacokinetic studies, this research provided a comprehensive analysis of the differences between the two formulations. Pharmacokinetic results showed that, compared to RFX-S, RFX-NS had a significantly reduced apparent volume of distribution (Vz/F: 856.02 ± 274.00 vs. 1404.17 ± 285.1 mL/kg, *p* < 0.01), a shorter elimination half-life (t_1/2_: 4.05 ± 1.18 vs. 5.32 ± 0.94 days, *p* < 0.05), and a relative bioavailability of 128.98%. These findings suggest that RFX-NS is an improved novel formulation superior to the conventional suspension, as it enhances drug efficacy and efficiency by improving drug dissolution and absorption.

## Simple Summary

This study aimed to establish a more sensitive and reliable quantitative detection method for rafoxanide in sheep plasma and apply it to an 84-day long-term pharmacokinetic study comparing a nanosuspension with a conventional suspension. The results showed that RFX-NS had a smaller particle size, a more uniform distribution, a higher Zeta potential, and better in vitro dissolution. Pharmacokinetic analysis revealed that RFX-NS had a smaller volume of distribution, a shorter elimination half-life, and a relative bioavailability of 128.98%. This demonstrates that RFX-NS is a superior novel formulation capable of improving drug dissolution and absorption.

## 1. Introduction

Ruminants are a crucial component of global agriculture and ecosystems, serving as an important source of products such as meat, milk, and hides for humans. However, parasitic diseases are one of the most serious problems in ruminant farming [1,2]. Liver flukes, lungworms, *Haemonchus contortus* (barber’s pole worm), and hookworms are all common parasitic infections in ruminants [3,4,5]. Once parasitized, ruminants gradually become emaciated, and in severe cases, may develop toxemia or secondary diseases. The severe decline in production performance—manifested as weight loss, reduced milk yield, reproductive disorders, deterioration in meat and leather quality, and even acute death—results in significant economic losses [6,7,8,9]. Fasciolosis, a zoonotic parasitic disease caused by the liver fluke *Fasciola hepatica* parasitizing the bile ducts of ruminants, is highly contagious and can often spread rapidly through water sources, infecting other animals and even humans [10,11]. Existing antiparasitic drug formulations for ruminants each have their own drawbacks: tablets are costly and inconvenient to administer; powders require mixing with feed, making precise dosing difficult; injectable formulations are inconvenient and require technical skill; and conventional suspensions are prone to sedimentation, leading to uneven drug content. Moreover, these common formulations typically require multiple administrations, which complicates management and use in large-scale farming operations. Therefore, this study aims to identify an ideal antiparasitic drug dosage form that can effectively control parasitic diseases while also offering high economic value and convenience.

Rafoxanide (RFX) is a halogenated salicylanilide anthelmintic that has long been widely used as a highly effective broad-spectrum antiparasitic agent [12,13,14]. In addition to Fasciola hepatica, RFX also exhibits potent efficacy against various gastrointestinal nematodes, such as Haemonchus contortus, Ostertagia spp., Bunostomum spp., and certain tapeworms [15]. Literature indicates that rafoxanide exhibits a notable propensity for self-association in aqueous media, whereby molecules form aggregates through non-covalent interactions such as π-π stacking and hydrophobic effects. This self-association behavior is one of its important physicochemical properties [16]. This characteristic is a critical factor influencing its solubility, crystallization, and formulation stability. Unlike other broad-spectrum anthelmintics, RFX directly attacks the final step of energy metabolism—the mitochondrial respiratory chain. Its parasiticidal mechanism lies in its action as an uncoupler on mitochondria, disrupting the proton gradient across the inner mitochondrial membrane. This causes energy to dissipate as heat, preventing the effective generation of ATP, and leading to parasite death due to energy exhaustion [17,18,19]. Resistance to benzimidazoles and macrocyclic lactones often arises from point mutations in the genes encoding the target site proteins or channel subunits, leading to reduced drug-binding affinity. For parasites under RFX pressure, however, it is difficult to alter the entire structure and function of mitochondria through a single gene mutation without affecting their own survival; often, coordinated variations in multiple genes are required to reduce drug-binding affinity. Precisely because of this, RFX exerts potent lethal effects against both adult and juvenile stages of Fasciola hepatica, and can counteract or delay the development of drug resistance, maintaining good therapeutic efficacy.

Commercially available dosage forms of RFX include powders, tablets, and suspensions. Powders are administered by mixing with feed, a method that places extremely high demands on the homogeneity of feed mixing and the accuracy of feed intake control; otherwise, it easily leads to either insufficient or excessive individual drug intake. Tablets are administered via individual oral dosing, primarily used in dogs for treating babesiosis; due to their production costs, they are seldom employed for ruminants. Suspensions are administered via oral drenching. Because conventional suspensions have overly large drug particles, they are highly prone to sedimentation, necessitating thorough shaking and rapid administration before use. This imposes relatively high labor cost demands for large-scale farming operations. However, RFX is a poorly water-soluble drug [16]. In contrast, a suspension can disperse solid drug particles in an aqueous solution, significantly enhancing its stability and solubility. When combined with emulsification technology, it can be formulated into a dispersed system with particle sizes in the micrometer range, thereby achieving penetrating targeted effects. If further nano-sized, its penetration capability can be significantly enhanced, achieving passive targeting effects, improving absorption, and increasing bioavailability. Studies have indicated that an ideal antiparasitic drug should possess sustained-release and long-acting properties [20] to combat the prolonged life cycle of parasites. Sustained-release is precisely one of the key characteristics and core advantages of nanosuspensions as an advanced drug delivery system: after nano-sizing, the total surface area of the drug increases exponentially, and the drug release rate is directly proportional to the dissolution surface area [21].

Currently, literature reports indicate that methods for detecting RFX plasma concentrations primarily rely on high-performance liquid chromatography (HPLC) [22,23,24,25,26]. While these methods offer a certain degree of reliability, they still exhibit limitations in terms of sensitivity, selectivity, and analytical throughput. For the nano-sized ionic rafoxanide suspension targeted in this study, it is evident that a more precise detection method is required to meet the demands for accurate quantification of trace drug levels in complex biological matrices. This study has established a Liquid Chromatography-Tandem Mass Spectrometry (LC-MS/MS) detection method with a lower limit of detection and higher sensitivity for quantifying RFX in sheep. This method combines the high separation efficiency and sensitivity of chromatography with the high specificity of mass spectrometry, significantly lowering the detection limit, improving analytical efficiency, and effectively avoiding matrix interference. The establishment of this method provides substantial reference value for promoting in-depth research and future clinical application of this drug.

Based on the theoretical foundations and analytical techniques described above, this study aims to establish a more sensitive and reliable detection method for the quantitative determination of rafoxanide (RFX) in sheep plasma. This method was applied to an 84-day long-term comparative pharmacokinetic trial in sheep, comparing rafoxanide nanosuspension (RFX-NS) with rafoxanide conventional suspension (RFX-S). The study integrated the characterization of the formulations and in vitro dissolution evaluation to comprehensively analyze the pharmacokinetic differences between the nanonized formulation and the conventional formulation in sheep, thereby elucidating the advantages of the nanosuspension dosage form.

## 2. Materials and Methods

### 2.1. Animals

Sixteen healthy sheep, aged 6–12 months and weighing 25–35 kg, were enrolled in this study. Exclusion criteria included a history of medication within the previous month or failure to meet the specified weight/age range. Each animal was identified with an ear tag and acclimatized for 7 days in a clean, quiet environment prior to the experiment. During acclimatization, the sheep were provided with a fixed amount of drug-free feed and water ad libitum daily. The animals were then randomly assigned to two groups (*n* = 8 per group) using a computer-generated randomization sequence produced by statistical software (SPSS 26.0). To control for potential sex-related variations, block randomization was performed with sex as a stratification factor, resulting in balanced groups each containing 4 rams and 4 ewes. The study protocol was reviewed and approved by the Animal Welfare and Ethics Committee of the Lanzhou Institute of Animal Science and Veterinary Medicine, Chinese Academy of Agricultural Sciences (Approval No.: 2026-31).

### 2.2. Chemicals, Reagents, and Laboratory Materials

RFX (Batch No.: CDAA-580059, purity ≥ 99.2%) was purchased from Shanghai ANPEL Laboratory Technologies Inc. (Shanghai, China). Its corresponding internal standard, Niclosamide (NIC) (Batch No.: G1038693, purity ≥ 99.0%), was provided by Dr. Ehrenstorfer GmbH (Augsburg, Germany). LC-MS grade methanol (Batch No.: 203398, purity: 99.9%) was obtained from Merck KGaA (Darmstadt, Germany). LC-MS grade formic acid and ammonium formate were purchased from Thermo Fisher Scientific (Waltham, MA, USA). Ultrapure water (Batch No.: 120212TS) was supplied by Wahaha Group Co., Ltd. (Hangzhou, China). Heparin sodium solution (Batch No.: 0828A23, concentration: 0.1%) was acquired from Beijing Ruigen Biotechnology Co., Ltd. (Beijing, China). RFX-NS was provided by Shandong Dezhou Shenniu Pharmaceutical Co., Ltd. (Dezhou, China).

Major instruments: Sartorius BSA223S electronic balance (Sartorius AG, Göttingen, Germany); SI Vortex-Genie 2 variable-speed vortex mixer (Scientific Industries, Inc., Bohemia, NY, USA); Eppendorf research^®^ plus single-channel pipettes (Eppendorf AG, Hamburg, Germany); Labconco RapidVap sample evaporation system (Labconco Corporation, Kansas City, MO, USA); AB Sciex 5500 ultra-performance liquid chromatography-tandem mass spectrometry system (SCIEX, Framingham, MA, USA); Malvern Zetasizer Nano ZS ZEN3600 Laser Dynamic Light Scattering Instrument (Malvern Panalytical Ltd., Malvern, UK); Sigma 300 Scanning Electron Microscope (SEM) (Carl Zeiss AG, Jena, Germany); Agilent 1290 Infinity High-Performance Liquid Chromatography System (Agilent Technologies, Inc., Santa Clara, CA, USA); SevenCompact™ S220 pH Meter (Sartorius AG, Göttingen, Germany); Pharma-Test PTWS610 Dissolution Tester (Pharma Test Apparatebau AG, Hainburg, Germany).

Data acquisition and processing software: Analyst 1.7.2 (SCIEX, Framingham, USA) software was used for mass spectrometry data acquisition; SciexOS 2.0.1 (SCIEX, Framingham, MA, USA) software for quantitative analysis; Microsoft Office Excel 2021 for calculating standard deviation (SD), mean, and coefficient of variation (C.V.). Pharmacokinetic parameter analysis and non-compartmental modeling were performed using Phoenix 8.1.0 software. Statistical significance analysis was conducted using SPSS Statistics 26.

### 2.3. Characterization of Suspensions

#### 2.3.1. Particle Size, PDI and Zeta Potential Measurement

Particle Size and PDI: Measured by laser dynamic scattering. The volume average particle size was recorded, and the uniformity of particle size distribution was evaluated by PDI. A PDI value typically less than 0.3 indicates a relatively narrow distribution and a homogeneous system.

Zeta Potential: Measured by electrophoretic light scattering in a dedicated electrode cell, and the average Zeta potential value was recorded. A higher absolute value (with a common reference threshold greater than ±30 mV) indicates stronger electrostatic repulsion between particles, leading to a more stable system that is less prone to aggregation.

#### 2.3.2. Morphological Analysis

The raw material powder of rafoxanide was directly sprinkled onto conductive adhesive, followed by gold sputtering for observation. In contrast, both RFX-S and RFX-NS were dropped onto silicon wafers, dried, and subsequently coated with gold before examination. A systematic magnification strategy was adopted for image acquisition. For the raw material and the conventional suspension, scanning electron microscopy (SEM) was employed to capture morphological features sequentially at scale bars of 50 μm, 20 μm, 10 μm, and 5 μm. To clearly resolve the nanoparticles, additional high-resolution images at scale bars of 1 μm and 500 nm were acquired for the nanosuspension. All operations were performed under a constant accelerating voltage of 5 kV to ensure the comparability of the results.

#### 2.3.3. In Vitro Dissolution Study

By screening the dissolution media, rotation speed, enzymes, and surfactants for the two types of suspensions, the optimal dissolution medium, rotation speed, enzyme, and surfactant were determined. Under the selected optimal conditions, with a dissolution medium volume of 900 mL and the dissolution temperature set at 37 ± 0.5 °C, RFX-NS and RFX-S were weighed and added to the dissolution vessels, and the timer was started. Samples of 5 mL were taken at 15, 30, 45, and 60 min, with 5 mL of pre-warmed dissolution medium replenished immediately. The collected samples were filtered through a 0.45 μm organic-phase microporous membrane and analyzed using HPLC to determine the content and dissolution rate. Dissolution curves were plotted and evaluated.

#### 2.3.4. Stability Study

RFX-NS and RFX-S were separately dispensed and stored under sealed, light-protected conditions at 4 °C and 25 °C ± 2 °C for three months. At the end of the three months, samples were taken to observe and determine their appearance, particle size, sedimentation volume ratio, redispersibility, PDI, Zeta potential, and drug content. The trends of these indicators over time were analyzed to preliminarily evaluate the stability of the formulation.

### 2.4. Preparation of Stock Solutions

The drug reference standards were dissolved in methanol to prepare working solutions of RFX and NIC. The working solutions were then added to blank sheep plasma to prepare standard plasma samples and quality control (QC) samples. The calibration range for RFX was 2.5–800 ng/mL. Using sheep plasma as the blank, QC samples were prepared at the lower limit of quantification (LLOQ: 2.5 ng/mL), low QC (LQC: 8 ng/mL), medium QC (MQC: 80 ng/mL), and high QC (HQC: 750 ng/mL) concentrations.

### 2.5. Sample Preparation

A 500 µL aliquot of sheep plasma was placed into a 10 mL centrifuge tube, followed by the addition of 10 µL of working solution (300 ng/mL NIC) and 500 µL of phosphate-citrate buffer (pH = 6). After vortexing, 5 mL of ethyl ether was added. The mixture was vortexed again, subjected to an ultrasonic water bath (4 °C, 10 min), and then allowed to stand. The upper ether extract was then decanted and transferred to another 10 mL centrifuge tube. The remaining sample in the original tube underwent a second extraction with 5 mL of ethyl ether, repeating the vortexing, sonication, standing, and decanting steps. The two ether extracts were combined. The combined extract was evaporated to dryness. The residue was reconstituted in 500 µL of LC-MS grade methanol by vortexing and sonication. The resulting solution was then filtered through a 0.22 µm membrane prior to concentration analysis.

### 2.6. LC-MS/MS Instrumentation and Setting

The analysis was performed using an LC-30AD ultra-high performance liquid chromatograph (Shimadzu, Kyoto, Japan) coupled to a QTRAP^®^ 5500 triple quadrupole mass spectrometer (AB Sciex, USA) equipped with an electrospray ionization (ESI) source. Detection was carried out in multiple reaction monitoring (MRM) mode with negative ion detection. The ion source parameters were set as follows: ion spray voltage (IS): +5500 V, ion source temperature (TEM): 550 °C, nebulizer gas pressure (GS1): 50 psi, auxiliary gas pressure (GS2): 55 psi, curtain gas pressure: 35 psi, and dwell time: 150 ms.

Chromatographic separation was achieved on an ACQUITY UPLC BEH C18 column (1.7 µm, 2.1 × 100 mm) maintained at 30 °C. A gradient elution program was employed with a flow rate of 0.3 mL/min and an injection volume of 5 µL. The mobile phase consisted of (A) 0.1% formic acid and 10 mmol/L ammonium formate in water, and (B) methanol. The optimized gradient program was as follows: 30% to 100% B from 0 to 5.0 min, 100% B from 5.0 to 8.0 min, 100% to 30% B from 8.0 to 8.1 min, and 30% B from 8.1 to 10.0 min.

### 2.7. Method Validation

The method validation was conducted in accordance with the bioanalytical method validation guidelines issued by the European Medicines Agency (EMA) and the U.S. Food and Drug Administration (FDA). The validated parameters included linearity, matrix effect, accuracy and precision, stability, and dilution integrity.

#### 2.7.1. Selectivity and Specificity

Plasma samples were prepared from healthy experimental sheep: these included blank plasma samples, plasma samples spiked with only the RFX standard, plasma samples spiked with only the internal standard (IS), plasma samples spiked with both the RFX standard (at a concentration of 100 ng/mL) and the internal standard NIC (300 ng/mL), and post-administration plasma samples. This procedure was designed to validate the selective interference of plasma matrix or other co-existing substances on the detection of the target drug, ensuring the specificity of the chromatographic peak for the target drug in the plasma matrix.

#### 2.7.2. Linearity

The standard curve for the sample was prepared according to the method described in Section 2.3. The concentration of the analyte served as the abscissa (*x*-axis), while the ratio of the analyte peak area to the corresponding internal standard peak area served as the ordinate (*y*-axis). Weighted least-squares linear regression analysis was performed using SciexOS 2.0.1 software to calculate the regression equation and the correlation coefficient (R^2^), and to plot the standard curve.

#### 2.7.3. Accuracy and Precision

Four sets of plasma quality control (QC) samples were prepared with specified concentrations at the lower limit of quantification (LLOQ), low, medium, and high levels, with six replicates at each concentration. A series of standard plasma samples were also prepared. All samples were processed according to the method described in Section 2.4 and subsequently injected for analysis. The analysis was performed in three independent batches on three separate days. The actual concentrations of RFX in the four QC levels were calculated using the internal standard method. For QC samples, the required accuracy (expressed as percent nominal) for both within-run and between-run assessments was 85–115% (80–120% for LLOQ). To evaluate precision, the relative standard deviation (RSD) of the measured values (RSD = standard deviation/mean × 100%) was used. The RSD for both within-run and between-run precision was required to be ≤15% (≤20% for LLOQ).

#### 2.7.4. Matrix Effect

The matrix effect was determined by the ratio of the analyte peak area in the presence of matrix to the analyte peak area in the absence of matrix. The matrix factor for the analyte, corrected for the matrix factor of the internal standard, yields the internal standard-normalized matrix factor. The coefficient of variation for the normalized matrix factor should be less than 15%.

The matrix effect factor (MF) is defined as the ratio of the analyte response (peak area) in the biological matrix to the analyte response in the pure solution. On this basis, the internal standard-normalized matrix effect factor (MFi) is obtained by dividing the absolute matrix factor of the analyte by the absolute matrix factor of the internal standard. This normalization corrects for the matrix effect inherent to the internal standard itself, thereby adjusting for matrix interference.

#### 2.7.5. Stability

The stability of QC plasma samples at low and high concentrations was evaluated under conditions of room temperature, repeated freeze–thaw cycles, and long-term storage at −80 °C. Two levels of standard plasma samples were prepared. For each concentration level, three independent batches were prepared, with eight replicates per batch. A series of standard plasma samples were also prepared. After the initial analysis, the data were recorded. Subsequently, the three batches of samples were subjected to different treatments: the room-temperature batch was kept at room temperature for 24 h; the repeated freeze–thaw batch was stored at −20 °C and subjected to three complete freeze–thaw cycles; and the −80 °C batch was stored at −80 °C for 7 days. After each treatment, the batches were injected and analyzed, and the data were recorded.

#### 2.7.6. Carryover

High-concentration standard plasma samples, lower limit of quantification (LLOQ) concentration samples, and blank plasma samples were prepared separately. Immediately after the analysis of the high-concentration QC plasma samples, the blank and LLOQ samples were analyzed. The peak area of the residual analyte in the blank sample was calculated and compared to that of the LLOQ sample. The residual amount of the analyte in the blank sample should not exceed 20% of the LLOQ level, and should not exceed 5% of the internal standard.

#### 2.7.7. Dilution Integrity

The sample was diluted 1:20 with blank plasma, and the analyte was spiked into the matrix at a concentration exceeding the upper limit of quantification (ULOQ). This procedure demonstrated dilution integrity. The accuracy and precision of the assay were confirmed to be within ±15%. Furthermore, the reliability of the dilution was verified to cover the concentration range of the test samples.

### 2.8. Pharmacokinetics

#### 2.8.1. Experimental Design and Sample Collection

The sheep were grouped and managed as described in Section 2.1. Eight sheep in Group 1 were orally administered a single dose of Rafoxanide Nanosuspension (RFX-NS) at 12 mg/kg, while eight sheep in Group 2 were orally administered a single dose of Rafoxanide conventional suspension (RFX-S) at the same dosage. Before dosing, a 5 mL blank blood sample was collected from the jugular vein and placed in a sodium heparin anticoagulant tube. After administration, 5 mL blood samples were collected at the following time points: 1/12, 1/6, 1/4, 1/3, 1/2, 1, 2, 3, 5, 6, 7, 8, 9, 11, 12, 13, 14, 21, 28, 35, 42, 56, 70, and 84 days. All blood samples were centrifuged at 3000 rpm for 15 min to separate the plasma, and the supernatants were stored at −20 °C until analysis. The plasma concentration–time curve for each sheep was plotted, and both individual and mean plasma concentration–time curves were obtained.

#### 2.8.2. Data Processing and Pharmacokinetic Parameter Analysis

The plasma RFX concentration-time data were analyzed using Phoenix 8.1.0 software. Pharmacokinetic parameters were calculated using a non-compartmental analysis (NCA) method. The elimination rate constant (λz) was determined by log-linear regression of at least the last 3 data points on the terminal phase of the plasma drug concentration-time curve exhibiting a stable mono-exponential decline. The terminal elimination half-life (t_1_/_2_) was calculated using the formula: 0.693/λz.

The primary parameters calculated included: time to reach the maximum plasma concentration (T_max_), maximum plasma concentration (C_max_), area under the plasma concentration-time curve from time zero to the last quantifiable concentration (AUC_0–t_), area under the plasma concentration-time curve from time zero extrapolated to infinity (AUC_0–∞_), apparent volume of distribution (Vz/F), clearance (CL/F), and mean residence time (MRT). Other standard non-compartmental parameters were also calculated. The percentage of the AUC extrapolated to infinity (%AUCextrap) was calculated using the formula: %AUCextrap = [(AUC_0–∞_ − AUC_0–t_)/AUC_0–∞_] × 100%, to assess the contribution of the extrapolated portion to the total drug exposure. The relative bioavailability (F) of the nanosuspension relative to the conventional suspension was calculated as: F = (AUC_0–t_ of RFX-NS/AUC_0–t_ of RFX-S) × 100%.

#### 2.8.3. Rationale for Selection of Analytical Method

The non-compartmental analysis (NCA) method was selected primarily based on the following two considerations: First, the core objective of this study was to directly compare the key exposure and disposition parameters between the two formulations, RFX-NS and RFX-S, in order to evaluate their sustained-release characteristics and relative bioavailability. The NCA method, which does not rely on assumptions of a specific compartmental model structure, allows for the direct calculation of parameters from experimental data, providing intuitive results that facilitate inter-formulation comparison. Second, the pharmacokinetic processes of RFX in ruminants are complex, and its absorption and elimination may not conform to typical compartmental models. Preliminary analysis revealed issues such as poor goodness-of-fit or inconsistent model selection criteria when attempting to fit the data using compartmental models. To avoid introducing bias by imposing an inappropriate model, and to ensure the robustness and comparability of parameter estimates, the NCA method based on statistical moment theory was ultimately adopted.

### 2.9. Blinding

To reduce measurement bias, this study implemented a blinding procedure for the analysis of plasma samples. After collection, the samples were uniformly coded by researchers not involved in the grouping to conceal their group assignments. The personnel responsible for LC-MS/MS sample preparation, instrumental analysis, and initial data processing were blinded to the treatment groups of the samples. Pharmacokinetic parameter calculation was performed by another researcher under blinded conditions. Unblinding and statistical comparisons between groups were conducted only after all data analyses were completed.

## 3. Results

### 3.1. LC-MS/MS Method Development

**Figure 1:**
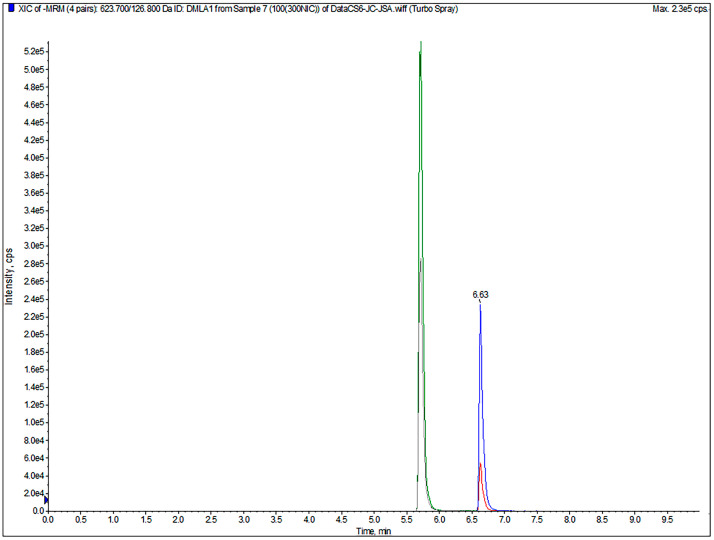
Chromatogram of standard working solutions containing 100 ng/mL Rafoxanide (RFX, blue trace) and 300 ng/mL Niclosamide (NIC, green trace).

Representative chromatograms are shown in Figure 1. Table 1 presents the MRM transitions and MS settings applied to the analytes in this method.

### 3.2. Characterization of the Suspensions

#### 3.2.1. Particle Size, PDI and Zeta Potential

As shown in Table 2, dynamic light scattering measurements revealed that the average particle size of the rafoxanide nanosuspension was 484.93 ± 43.11 nm, with a polydispersity index (PDI) of 0.06 ± 0.07, indicating a narrow particle size distribution and good system homogeneity. In contrast, for the conventional suspension, a portion of the particles exceeded the effective detection range of the laser particle size analyzer due to excessively large size and broad distribution, preventing accurate determination of the PDI. The portion that was measurable showed a particle size of 2379.67 ± 121.71 nm with a PDI of 0.93 ± 0.10, indicating a broad particle size distribution and poor system homogeneity.

Table 3 presents the absolute Zeta potential value of RFX-NS was 43.59 ± 0.67 mV. This relatively high absolute value indicates that the particle surface carries a strong negative charge, resulting in significant electrostatic repulsion between particles, which is favorable for the long-term physical stability of the nanosized dispersion system. In contrast, the conventional RFX-S exhibited a lower absolute Zeta potential of 38.10 ± 0.55 mV, indicating relatively poorer stability.

#### 3.2.2. Morphology

**Figure 2:**
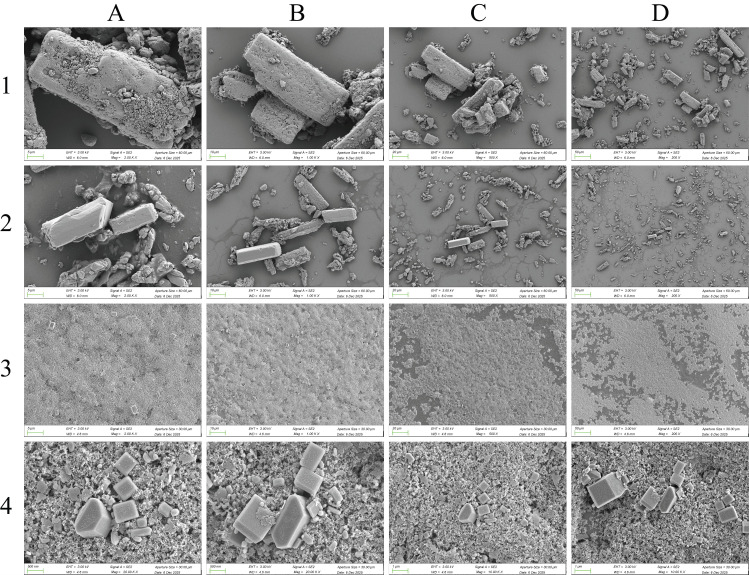
(**A1**–**D1**): Morphology of the original rafoxanide powder at 50 μm, 20 μm, 10 μm, and 5 μm scales; (**A2**–**D2**): Morphology of RFX-S at 50 μm, 20 μm, 10 μm, and 5 μm scales; (**A3**–**D3**): Morphology of RFX-NS at 50 μm, 20 μm, 10 μm, and 5 μm scales; (**A4**,**B4**): Morphology of RFX-NS at 1 μm scale; (**C4**,**D4**): Morphology of RFX-NS at 500 nm scale.

As shown in Figure 2, scanning electron microscopy (SEM) images at different magnifications reveal significant differences in the micromorphology among the sample groups. The rafoxanide active pharmaceutical ingredient (Figure 2(A1–D1)) displays a typical irregular blocky crystalline structure. The particles are relatively large (micrometer-scale) with a rough surface, distinct edges, and noticeable agglomeration. RFX-S (Figure 2(A2–D2)) shows a particle morphology similar to the raw material, retaining the elongated or blocky crystal characteristics. Although subjected to dispersion, the particle size remains in the micrometer range without significant reduction. In contrast, RFX-NS (Figure 2(A3–D4)) exhibits a marked change in morphology: at lower magnification (Figure 2(A3–D3)), it presents a dense, film-like structure with blurred particle boundaries, while at higher magnification (Figure 2(A4–D4), scale bars: 1 μm, 500 nm), the particles are clearly refined to the nanometer scale, appearing as irregular polyhedra or near-spherical shapes. The surface is relatively smooth, the distribution is uniform, and no significant agglomeration is observed, confirming the effectiveness of the nanosizing process.

#### 3.2.3. In Vitro Dissolution Profiles

**Figure 3:**
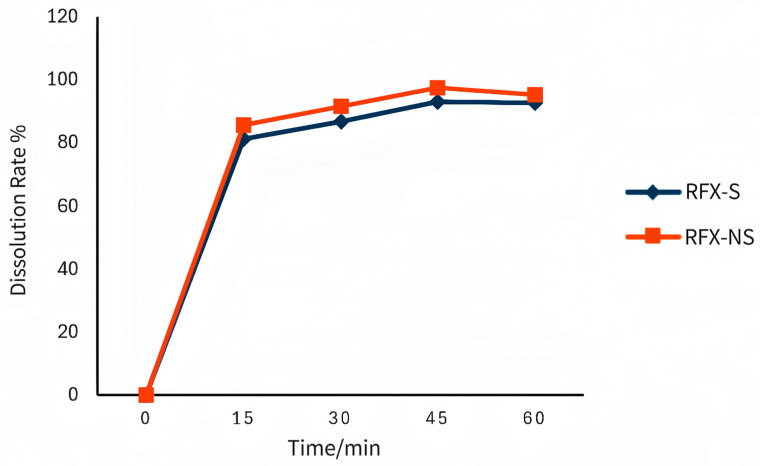
Dissolution Profile of RFX-S and RFX-NS.

Through the screening of dissolution media, rotation speed, and surfactants for RFX-NS and RFX-S, phosphate buffer with a pH of 8.0 was identified as the optimal dissolution medium, and a rotation speed of 75 r/min was determined to be the best. Using phosphate buffer (pH 8.0) as the dissolution medium under conditions of 75 r/min and a temperature of 37 ± 0.5 °C, the dissolution rates of the two rafoxanide suspensions were calculated as shown in Table 4, and the dissolution profiles are plotted in Figure 3. The results show that under the established dissolution method, the maximum dissolution rate of RFX-NS reached 97.6%, followed by RFX-S with a dissolution rate of 93.1%.

#### 3.2.4. Stability Study Results

In a 3-month stability study, rifaximin nanosuspension (RFX-NS) demonstrated significantly superior physical and chemical stability compared to the conventional suspension (RFX-S).

In terms of physical stability, RFX-NS remained uniform after storage at 4 °C and 25 °C, with no significant sedimentation observed. It exhibited a high sedimentation volume ratio and good redispersibility. The initial particle size was 484.93 ± 43.11 nm with a PDI of 0.06 ± 0.07. After 3 months, the particle size remained at the nanoscale with a narrow distribution (4 °C: 599.00 ± 28.85 nm, PDI = 0.26 ± 0.03; 25 °C: 567.83 ± 48.15 nm, PDI = 0.24 ± 0.01). In contrast, RFX-S showed obvious phase separation and sedimentation. Its initial particle size was already in the micrometer range (2379.67 ± 121.71 nm, PDI = 0.93 ± 0.10). Although changes after storage were not significant, it remained in the micrometer range throughout, and its stability was far inferior to the nano-system (4 °C: 2579.67 ± 111.96 nm, PDI = 1.00 ± 0.00; 25 °C: 2535.67 ± 217.83 nm, PDI = 0.95 ± 0.06).

The absolute Zeta potential of the rifaximin nanosuspension was initially 43.59 ± 0.67 mV. After 3 months of storage at 4 °C and 25 °C, the values were 40.22 ± 1.70 mV and 40.49 ± 0.60 mV, respectively, with absolute values still above 40 mV. These values were consistently and significantly higher than those of the conventional suspension, which had an initial absolute value of 38.10 ± 0.55 mV, decreasing to 36.85 ± 0.66 mV and 36.97 ± 0.93 mV after storage at 4 °C and 25 °C, respectively. A higher absolute Zeta potential indicates stronger electrostatic repulsion between particles, suggesting superior electrostatic stability for the nanosuspension, which is one of the key reasons for its better physical storage stability.

The drug content retention rate also confirmed the higher stability of the NS. The initial drug content of RFX-NS was close to the theoretical value (100.11 ± 1.49%) and remained at a high level after 3 months of storage (4 °C: 94.38 ± 2.51%; 25 °C: 96.15 ± 3.12%). In contrast, the initial drug content of RFX-S was lower (93.13 ± 1.22%) and decreased further after storage (4 °C: 88.33 ± 1.28%; 25 °C: 89.95 ± 1.69%).

### 3.3. Method Validation

#### 3.3.1. Selectivity and Specificity

**Figure 4:**
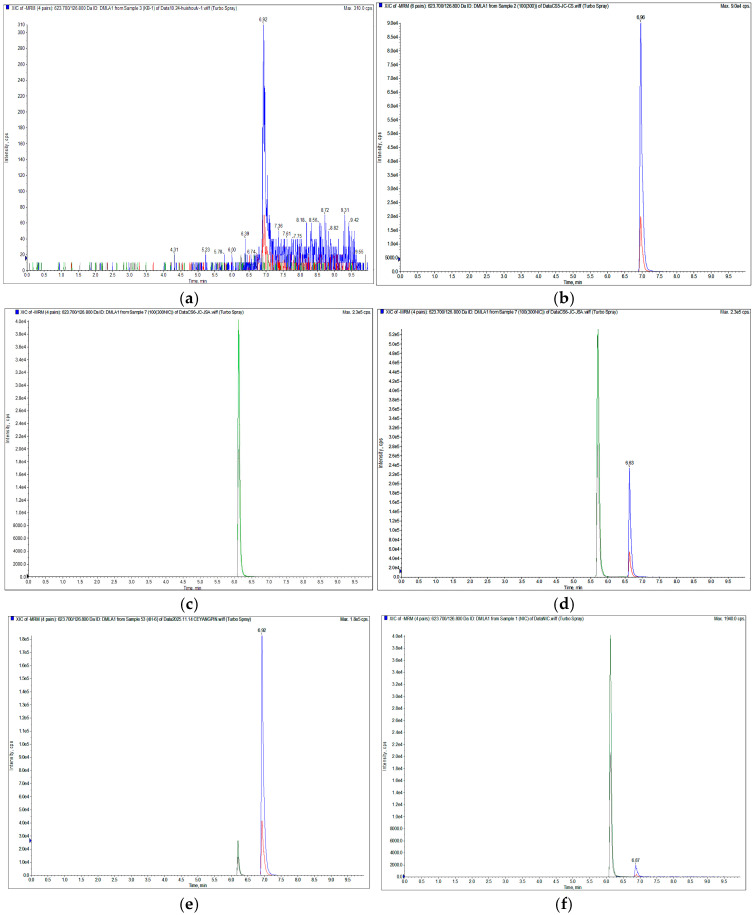
Chromatograms of (**a**) a blank sheep plasma sample, (**b**) a sheep plasma sample spiked with the RFX standard only, (**c**) a sheep plasma sample spiked with the NIC standard only, (**d**) a plasma sample spiked with RFX (at a concentration of 100 ng/mL) and the internal standard NIC (300 ng/mL), (**e**) a plasma sample collected from the first sheep 4 h after oral administration of rafoxanide nanosuspension (RFX-NS), and (**f**) a plasma sample collected from the first sheep 4 h after oral administration of conventional Rafoxanide Suspension (RFX-S). (The green chromatogram corresponds to the internal standard NIC, and the blue chromatogram corresponds to the drug RFX).

As shown in Figure 4, panel (a) represents blank sheep plasma; panel (b) represents sheep plasma spiked with rafoxanide (RFX) standard only; panel (c) represents sheep plasma spiked with the internal standard niclosamide (NIC) only; panel (d) represents sheep plasma spiked with both RFX and NIC; panel (e) represents a plasma sample collected 4 h after administration of RFX-NS; and panel (f) represents a plasma sample collected 4 h after administration of RFX-S. The analytical results demonstrate that both RFX and NIC exhibit good peak shapes with sufficient resolution, and their respective retention times do not interfere with each other, ensuring analytical accuracy. The detection results further indicate that endogenous components in the plasma do not cause any interference with the quantification of RFX, and the baseline remains stable. This confirms that the analytical method possesses high specificity and is capable of accurately and reliably determining the concentration of RFX in plasma.

#### 3.3.2. Linearity

**Figure 5:**
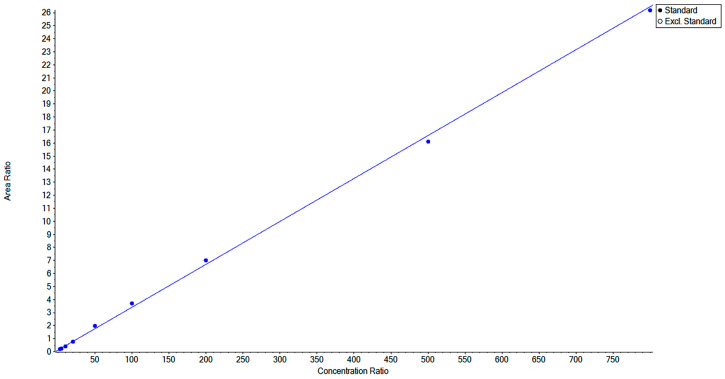
Standard curve of RFX.

**Figure 6:**
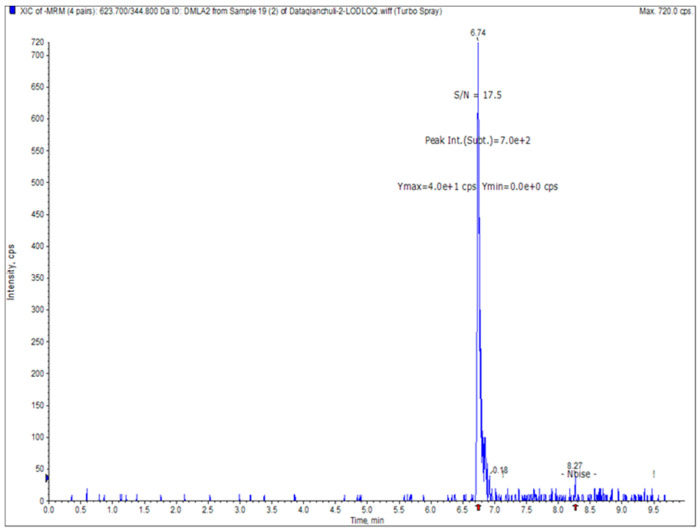
Plasma sample at the lower limit of quantification (LLOQ).

The standard curve was constructed with the ratio of chromatographic peak areas of the target compound RFX to the internal standard NIC as the ordinate (*y*-axis) and the mass concentration of RFX as the abscissa (*x*-axis). The standard curve is shown in Figure 5. The resulting regression equation and correlation coefficient were calculated as follows: y = 0.03292x + 0.11852 (r = 0.99914, r^2^ = 0.99827). As shown in Figure 6. The limit of quantification (LLOQ) for RFX was determined to be 2.5 ng/mL when the signal-to-noise ratio (S/N) exceeded 10.

#### 3.3.3. Accuracy and Precision

The within-day and between-day precision and accuracy for all quality control (QC) samples met the specified criteria. The accuracy (93%) and precision for the lower limit of quantification (LLOQ, 2.5 ng/mL) were within acceptable limits, and the deviation for all QC samples was within 15%. The relevant data are presented in Table 5.

#### 3.3.4. Matrix Effect

Standard samples without plasma and samples containing the sheep plasma matrix were prepared at low and high QC concentrations, respectively, for analysis. The coefficients of variation for the normalized matrix factor of RFX (n = 8) at the low and high concentrations were 4.58% and 3.06%, respectively, meeting the requirement that the coefficient of variation for the normalized matrix factor should not exceed 15%. Therefore, the matrix effect can be considered negligible in this method.

#### 3.3.5. Stability

As shown in Table 6, the impact of three different storage and handling conditions on the stability of the analyte in plasma matrices at high and low QC concentrations was investigated. The coefficients of variation (RSD) for all conditions were within the specified acceptable limits. This indicates that the analyte remains stable under the tested conditions, meeting the requirements for reliable measurement.

#### 3.3.6. Carryover

Blank sheep plasma samples and high-concentration QC plasma samples were used. Immediately after injecting the high-concentration sample, a blank sample was injected for analysis. The average residual response of RFX in the blank plasma sample was 3.29% of the response at the lower limit of quantification (LLOQ), corresponding to 2.89% of the internal standard response in the monitoring mode. This meets the requirements that the residual response in the blank sample should not exceed 20% of the LLOQ response and 5% of the internal standard response.

#### 3.3.7. Dilution Integrity

The coefficient of variation for RFX after a 20-fold dilution was 1.66%. The results indicate that the dilution process did not affect the accuracy or precision of the RFX concentration determination.

### 3.4. Pharmacokinetics

**Figure 7:**
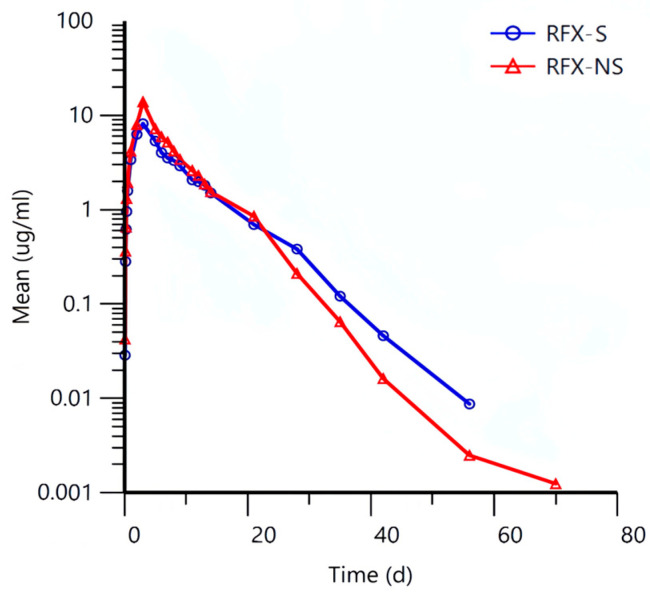
Plasma concentration-time profiles following administration of the two RFX suspensions (n = 8).

The established LC-MS/MS method was employed to analyze clinical plasma samples collected from 16 sheep following a single oral dose of RFX-S and RFX-NS, respectively. The plasma concentration-time profiles of RFX-S and RFX-NS are shown in Figure 7, and the corresponding pharmacokinetic parameters are presented in Table 7.

Figure 7 shows that the peak plasma concentration (Cmax) of RFX-NS is higher than that of RFX-S. The area under the plasma concentration-time curve (AUC) of RFX-NS is larger, indicating a greater total drug exposure and increased total absorption in vivo. The plasma concentration of RFX-NS declines more gradually, with a higher Cmax and a larger AUC, indicating greater total drug exposure.

The data in Table 7 shows that the t_1_/_2_ of RFX-NS is significantly shorter than that of RFX-S. The t_1_/_2_ of RFX-NS is 4.05 ± 1.18 d, while that of RFX-S is 5.32 ± 0.94 d, with *p* = 0.033. This indicates that RFX-NS is eliminated from the sheep’s body faster than RFX-S. The Vz/F of RFX-NS is significantly smaller than that of RFX-S. The Vz/F of RFX-NS is 856.02 ± 274.00 mL/kg, whereas that of RFX-S is 1404.17 ± 285.1 mL/kg, with *p* = 0.002 (*p* < 0.01, indicating a highly statistically significant difference). This suggests that RFX-NS remains more in the bloodstream compared to RFX-S. The area under the plasma concentration-time curve (AUC_0–t_ and AUC_0–∞_) of RFX-NS increased approximately 1.29-fold compared to that of RFX-S; The relative bioavailability of RFX-NS is 128.98%, indicating that its oral absorption efficiency is higher than that of RFX-S. All individual and mean group %AUCextrap values were less than 20% (the commonly accepted threshold is typically < 20–25%), confirming that the 84-day sampling period was sufficient to adequately characterize drug exposure, resulting in a highly reliable estimation of AUC(0–∞).

## 4. Discussion

### 4.1. Technical Analysis

Initially, analytes were injected into the mass spectrometer to optimize ionization parameters and detect fragmentation patterns. All analytes were detected in negative ion mode. Optimization of chromatographic conditions included selecting suitable mobile phases and columns. Studies have reported that adding a certain proportion of formic acid to the mobile phase can improve the ionization efficiency of analytes and enhance peak shape [22,23,27]. Building on this, this study also added a certain proportion of ammonium formate as a modification, which resulted in even better peak shapes. Literature commonly reports the use of methanol and acetonitrile as organic phases [22,24,25,26]. This study found that acetonitrile provided better elution than methanol. Therefore, mobile phase A: 0.1% formic acid—10 mmol/L ammonium formate aqueous solution and B: acetonitrile were selected. The ACQUITY UPLC BEH C18 column (1.7 µm, 2.1 × 100 mm) demonstrated excellent separation and symmetry. Furthermore, to separate the target compounds and achieve clear peaks, gradient elution mode was optimized. The optimal gradient elution program determined was: 30–100% B from 0 to 5.0 min, 100% B from 5.0 to 8.0 min, 100–30% B from 8.0 to 8.1 min, and 30% B from 8.1 to 10.0 min.

### 4.2. Sample Pretreatment

This study employed diethyl ether to extract rafoxanide (RFX) from sheep plasma. Research indicates that phenolic compounds tend to exist in their molecular form under acidic conditions (pH < pKa) [28]. RFX, containing a phenolic hydroxyl group, is a weak acidic phenolic compound with a pKa typically between 5–7. The ionization state of its phenolic hydroxyl group depends on the solution pH. Therefore, this study added a phosphate-citrate buffer solution (pH = 6) to create an acidic environment, increasing lipophilicity and facilitating the highest extraction recovery. Diethyl ether, a volatile organic solvent, is commonly used to extract hydrophobic compounds from biological samples. This allowed the RFX compound to be readily extracted from the sample matrix by diethyl ether. Existing methods often use HPLC for detection [23,24,25], which has limited sensitivity, often requiring large amounts of individual samples and multiple extractions with diethyl ether. After establishing the mass spectrometry method in this study, detection sensitivity was significantly improved, allowing a moderate reduction in individual sample amount and diethyl ether volume. This greatly reduced the waste of labor and time costs. Results showed good peak shapes, achieving equivalent extraction efficiency. The reduced usage of diethyl ether enhanced safety and lowered economic costs.

### 4.3. Formulation Characterization

RFX nanosuspension (RFX-NS) exhibited significantly superior physicochemical properties compared to RFX suspension (RFX-S). Dynamic light scattering and scanning electron microscopy (SEM) results showed that RFX-NS had an average particle size of 484.93 ± 43.11 nm and a polydispersity index (PDI) of 0.06 ± 0.07, indicating a uniform particle size distribution and nano-sized irregular polyhedrons. In contrast, RFX-S had a micron-scale particle size of 2379.67 ± 121.71 nm, a PDI of 0.93 ± 0.10, and was prone to sedimentation. More importantly, RFX-NS possessed a higher absolute Zeta potential value (43.59 ± 0.67 mV) compared to RFX-S (38.10 ± 0.55 mV), indicating stronger negative charges on the particle surfaces and greater interparticle electrostatic repulsion. This is a key factor ensuring the long-term physical stability of the dispersion system. In vitro dissolution experiments and a 3-month stability study further confirmed that RFX-NS outperformed the traditional suspension in terms of dissolution rate, system homogeneity, redispersibility, and drug content maintenance. These superior formulation characteristics provide the material basis for its distinct in vivo pharmacokinetic behavior.

During the 3-month storage period, RFX-NS demonstrated good long-term physical stability, specifically manifested as: after 3 months of storage at 4 °C and 25 °C, the particle size remained at the nano-level with a narrow distribution, no significant aggregation or sedimentation, and good redispersibility. Its absolute Zeta potential value remained high and was consistently higher than that of the traditional suspension. Drug content retention also confirmed the greater stability of RFX-NS. Notably, rafoxanide itself possesses self-association properties [16], which typically increase the risk of particle aggregation and precipitation in conventional suspensions, posing a challenge to their long-term stability. However, the nanotechnology and formulation process used for RFX-NS likely effectively inhibited further aggregation of drug nanoparticles due to self-association tendency by providing sufficient steric hindrance and the strong electrostatic repulsion conferred by the high Zeta potential. This indicates that the selected process successfully overcame the particle growth and aggregation issues caused by drug self-association, resulting in a stable nano-dispersion system during storage.

### 4.4. Pharmacokinetics

The 84-day comparative pharmacokinetic study conducted using the established LC-MS/MS method revealed significant differences between the two formulations. Compared to the non-nanomized traditional suspension (RFX-S), the rafoxanide nanosuspension (RFX-NS) exhibited the following characteristics: The apparent volume of distribution during the terminal phase (Vz/F) of RFX-NS was 1.64 times lower than that of RFX-S (856.02 ± 274.00 mL/kg vs. 1404.17 ± 285.1 mL/kg), being highly significantly smaller, indicating that RFX-NS remained more in the bloodstream compared to RFX-S. Rafoxanide itself has a strong affinity for plasma proteins. Combined with the characterization results of the two formulations, the nanoparticle size reduction via the nano-process may have further influenced its distribution pattern, leading to increased plasma concentration while reducing accumulation in non-target tissues. This not only helps enhance efficacy but may also lower potential toxicity risks to the organism.

Contrary to expectations, the elimination half-life (t1/2) of RFX-NS was 1.32 times shorter than that of RFX-S (4.05 ± 1.18 d vs. 5.32 ± 0.94 d), significantly smaller. This may reveal a more complex in vivo disposition process for the nano-formulation. Combining with the in vitro dissolution results of the two formulations, the dissolution rate of RFX-NS was 4.8% higher than that of RFX-S (97.6% vs. 94.8%). The faster dissolution rate of nanoparticles may have altered the characteristics of the terminal phase of the plasma concentration-time curve. The shortened t1/2 of RFX-NS could be attributed to the significant decrease in Vz/F, as t1/2 is proportional to Vz/F, and Vz/F is the numerator in the t1/2 calculation formula (t1/2 = 0.693 × Vz/CL). This conclusion aligns with the inference of reduced peripheral tissue exposure.

In addition, the Cmax in the RFX-NS group demonstrated considerable inter-individual variability, and some parameters did not reach significance, which may suggest that the sample size set in this study was insufficient. Confirmatory studies in the future should perform formal sample size estimation based on the preliminary experiment. Due to the limited sample size of this study (n = 8), formal subgroup or gender difference analyses were not conducted. Nevertheless, the consistent numerical trends observed across the pharmacokinetic parameters still reveal the potential advantages of the nanosuspension. For example, RFX-NS showed a trend towards prolonged T_max_, elevated C_max_, and an approximately 1.29-fold increase in AUC. Most importantly, the apparent volume of distribution (Vz/F), representing drug distribution, was significantly reduced, while the clearance (CL/F) also exhibited a clear downward trend. This pattern of “reduced volume of distribution and decreased clearance” aligns with an improved and more sustained absorption process, which may contribute to prolonging the drug’s residence time in the body.

### 4.5. Summary

In summary, although some inter-group comparisons did not reach the traditional threshold of statistical significance, multiple pharmacokinetic parameters synergistically presented consistent numerical trends that align with the theoretical mode of action for nano-formulations. These results strongly suggest that nanomization technology significantly altered the in vivo disposition behavior of rafoxanide, demonstrating clear potential in enhancing bioavailability (estimated relative bioavailability increased by 28.98%), achieving more sustained drug exposure, and potentially optimizing tissue distribution. This study provides important preliminary evidence and clear research directions for subsequent larger-scale confirmatory studies and further formulation optimization.

## 5. Conclusions

This study successfully established a sensitive and reliable LC-MS/MS method for the quantitative detection of RFX in sheep plasma. An 84-day comparative pharmacokinetic study conducted with this method demonstrated that RFX-NS, compared to RFX-S, possesses a smaller particle size, a more uniform distribution, a higher Zeta potential, and superior in vitro dissolution characteristics. Pharmacokinetic analysis revealed that RFX-NS showed a significantly reduced apparent volume of distribution, a shortened elimination half-life, and a relative bioavailability of 128.98% in sheep. These findings collectively demonstrate that RFX-NS is a novel and improved formulation. By enhancing drug dissolution and absorption, it can improve efficacy and therapeutic efficiency, holding potential as an ideal anthelmintic dosage form.

## Figures and Tables

**Table 1:** Mass Spectrometric Conditions for Each Analyte.

Analyte	Precursor Ion (*m*/*z*)	Product Ion (*m*/*z*)	Collision Energy (V)
Rafoxanide (RFX)	623.7	126.8 ^1^/344.81	−105/−48
Niclosamide (NIC)	325.1	171/289.21 ^1^	−36/−24

^1^ Quantitative ion used for quantification.

**Table 2:** Particle Size Distribution and Polydispersity Index (PDI) Determination.

Batch	Particle Size (nm)		Polydispersity Index (PDI)	
	RFX-S	RFX-NS	RFX-S	RFX-NS
1	2254.0	435.4	0.9692	0.0165
2	2388.0	514.0	0.8173	0.0174
3	2497.0	505.4	1.0000	0.1390

**Table 3:** Zeta potential determination.

Batch	Zeta Potential (mV)			
	RFX-S	Mean ± SD	RFX-NS	Mean ± SD
1	−38.32	−38.10 ± 0.55	−44.00	−43.59 ± 0.67
2	−38.51		−43.96	
3	−37.48		−42.82	

**Table 4:** In Vitro Dissolution of Rafoxanide Suspensions.

Preparation	Dissolution Rate (%)			
	15 min	30 min	45 min	60 min
RFX-S	81.2	86.9	93.1	92.5
RFX-NS	85.5	91.4	97.6	95.2

**Table 5:** Accuracy and Precision for the Determination of RFX in Sheep Plasma by Liquid Chromatography-Tandem Mass Spectrometry (LC-MS/MS).

Nominal Concentration (ng/mL)v	Measured Concentration (Mean ± SD, ng/mL)	Intra-Day (n = 8)		Inter-Day (n = 24)	
		Precision (RSD%)	Accuracy (%)	Precision (RSD%)	Accuracy (%)
	2.40 ± 0.14	96.05	5.85		
2.5	2.36 ± 0.12	94.20	5.30	93.98	5.46
	2.29 ± 0.11	91.70	4.74		
	7.12 ± 0.21	89.00	2.89		
8	7.47 ± 0.30	93.38	3.99	93.49	6.55
	7.85 ± 0.59	98.11	7.58		
	85.48 ± 1.80	106.84	2.10		
80	87.08 ± 1.56	108.84	1.79	107.49	2.41
	108.86 ± 1.97	106.80	2.97		
	737.88 ± 33.71	98.38	4.57		
750	760.25 ± 20.86	101.37	2.74	101.19	4.00
	778.63 ± 22.61	103.82	2.90		

**Table 6:** Stability of RFX in Sheep Plasma (n = 8).

Analyte	Nominal Concentration (ng/mL)	Short-Term (25 °C, 24 h)		Freeze–Thaw Stability 3 Cycles		Long-Term (−80 °C, 7 d)	
		Measured Concentration (ng/mL)	RSD (%)	Measured Concentration (ng/mL)	RSD (%)	Measured Concentration (ng/mL)	RSD (%)
Rafoxanide	8	7.03	2.26	6.94	1.31	6.89	0.91
	750	710.75	1.32	662.43	1.55	655.95	1.75

**Table 7:** Main Pharmacokinetic Parameters of the Two RFX Suspensions.

Parameters	Unit	RFX-S	RFX-NS	*p*-Value
t_1/2_	d	5.32 ± 0.94	4.05 ± 1.18	0.033 *
T_max_	d	2.63 ± 0.52	2.75 ± 0.46	0.619
C_max_	ug/mL	9.04 ± 2.54	14.00 ± 7.56	0.101
AUC _0–t_	d*ug/mL	66.83 ± 12.07	86.20 ± 25.13	0.07
AUC _0–∞_	d*ug/mL	66.95 ± 12.09	86.28 ± 25.13	0.07
%AUCextrap	%	0.18 ± 0.10	0.10 ± 0.06	0.067
AUMC	d*d*ug/mL	601.51 ± 194.78	657.66 ± 170.21	0.549
MRT	d	8.88 ± 1.58	7.77 ± 1.28	0.148
Vz/F	mL/kg	1404.17 ± 285.1	856.02 ± 274.00	0.002 **
CL/F	mL/d/kg	185.74 ± 42.07	148.65 ± 38.98	0.089
Relative Bioavailability	%	/	128.98	/

t_1/2_: Elimination half-life; T_max_: Time to reach maximum concentration; C_max_: Maximum (peak) concentration; AUC_0–t_: Area under the plasma concentration-time curve from time zero to the last measurable time point; AUC_0-∞_: Area under the plasma concentration-time curve from time zero extrapolated to infinity; AUCextrap: Area under the plasma concentration–time curve from the last quantifiable time point to infinity, as estimated by extrapolation (Clast/λz); AUMC: Area under the (first) moment curve; MRT: Mean residence time; Vz/F: Apparent volume of distribution during the terminal phase; CL/F: Apparent clearance. * *p* < 0.05, ** *p* < 0.01; “/” indicates that no data is available for calculation under this entry.

## Data Availability

The corresponding authors will make the data supporting this study available upon reasonable request.
